# A multicentre randomized controlled trial of an empowerment-inspired intervention for adolescents starting continuous subcutaneous insulin infusion - a study protocol

**DOI:** 10.1186/1471-2431-13-212

**Published:** 2013-12-20

**Authors:** Anna Lena Brorsson, Janeth Leksell, Gunnel Viklund, Anna Lindholm Olinder

**Affiliations:** 1 Department of Women’s and Children’s Health, Karolinska Institute and Hospital, Stockholm, Sweden; 2 School of Health and Social Studies, Högskolan Dalarna, Sweden; 3 Department of Medical Sciences, Uppsala University, Uppsala, Sweden; 4 Department of Clinical Science and Education, Karolinska Institute, Södersjukhuset, Stockholm, Sweden

**Keywords:** Type 1 diabetes, Continuous subcutaneous insulin infusion (CSII), Adolescence, Parental involvement, Person-centred care, Guided self-determination-Young (GSD-Y)

## Abstract

**Background:**

Continuous subcutaneous insulin infusion (CSII) treatment among children with type 1 diabetes is increasing in Sweden. However, studies evaluating glycaemic control in children using CSII show inconsistent results. The distribution of responsibility for diabetes self-management between children and parents is often unclear and needs clarification. There is much published support for continued parental involvement and shared diabetes management during adolescence. Guided Self-Determination (GSD) is an empowerment-based, person-centred, reflection and problem solving method intended to guide the patient to become self-sufficient and develop life skills for managing difficulties in diabetes self-management. This method has been adapted for adolescents and parents as Guided Self-Determination-Young (GSD-Y). This study aims to evaluate the effect of an intervention with GSD-Y in groups of adolescents starting on insulin pumps and their parents on diabetes-related family conflicts, perceived health and quality of life (QoL), and metabolic control. Here, we describe the protocol and plans for study enrolment.

**Methods/design:**

This study is designed as a randomized, controlled, prospective, multicentre study. Eighty patients between 12–18 years of age who are planning to start CSII will be included. All adolescents and their parents will receive standard insulin pump training. The education intervention will be conducted when CSII is to be started and at four appointments in the first 4 months after starting CSII. The primary outcome is haemoglobin A1c levels. Secondary outcomes are perceived health and QoL, frequency of blood glucose self-monitoring and bolus doses, and usage of carbohydrate counting. The following instruments will be used: Disabkids, ‘Check your health’, the Diabetes Family Conflict Scale and the Swedish Diabetes Empowerment Scale. Outcomes will be evaluated within and between groups by comparing data at baseline, and at 6 and 12 months after starting treatment.

**Discussion:**

In this study, we will assess the effect of starting an CSII together with the model of GSD to determine whether this approach leads to retention of improved glycaemic control, QoL, responsibility distribution and reduced diabetes-related conflicts in the family.

**Trial registration:**

Current controlled trials: ISRCTN22444034

## Background

### Diabetes

Type 1 diabetes is the most predominant form of diabetes among children in Sweden. The most common treatment is multiple daily injections (MDI), but treatment with continuous subcutaneous insulin infusion (CSII) is an alternative that is being used more frequently. In 2011, 42% of the children with type 1 diabetes in Sweden were treated using CSII [1]. In many children and adolescents, insulin requirements decrease transiently after they have been diagnosed with type 1 diabetes and insulin treatment has been initiated. The remission phase in type 1 diabetes is defined as an insulin requirement < 0.5 IU/kg with a haemoglobin A1c (HbA1c) of < 53 mmol/mol [2]. The recommended HbA1c target for children and adolescents is < 58 mmol/mol without increasing the number of hypoglycaemic episodes [2,3]. The Diabetes Control and Complications Trial (DCCT) and follow up data clearly indicate that poor glycaemic control during adolescence and young adulthood increases the risks of severe complications, such as nephropathy and retinopathy later in life [4,5]. In Sweden, there are clear, age-related differences in HbA1c results among young people with diabetes. After starting school (at age 7 years), HbA1c tends to increase with increasing age [6,7].

### Insulin pump treatment

CSII is currently the most physiological way to deliver insulin [8]. The clinical indications for starting CSII for children and adolescents are broad. Treatment with CSII is associated with an increased cost compared with MDI. However, some studies have shown that CSII is cost effective when it results in improved metabolic control and quality of life (QoL) [9-11]. Structured and intensive education on basic and specific requirements of insulin pump therapy is essential for the patient and their parents so that they become familiar with the pump and its features [12]. Studies evaluating glycaemic control in children using CSII have shown divergent results [13-17]. One explanation for deteriorated glycaemic control among adolescents treated with CSII is omitted bolus doses before meals [18,19]. This is mainly explained by loss of focus when the children forget to take their medication. The distribution of responsibility for diabetes self-management between children and parents is often unclear and needs to be clarified [20,21].

### Parental involvement

Adolescence is the period when responsibility for diabetes management should be transferred from the parents to the teenager [22]. There is much published support for continued parental involvement and shared diabetes management during adolescence [22-25]. Communication and family dynamic factors appear to be important variables for glycaemic outcomes in adolescents with diabetes. They are stronger predictors of glycaemic control than age, gender or insulin treatment regimen [26]. Adolescents starting CSII found coping with diabetes easier than adolescents using MDI, but there were no differences in QoL, depression and self-efficacy [16]. Viklund et al. showed that five categories are important for decision making competence: cognitive maturity, personal qualities, experience, social network and parental involvement. Teenagers describe how parental involvement can be constructive or destructive [24], and the challenge is to find a level that is comfortable for all involved [22]. Children who have many diabetes-related conflicts with parents have higher HbA1c compared with those who have fewer conflicts, and the quality of their relationships is a critical factor in diabetes self-management [27].

### GSD-Young

Self-management is an active and proactive process that involves activities and goals and that is continuously ongoing. It involves shared but shifting responsibility for diabetes care and decision making between the child and their parents, and it is a process that also involves health care staff. It is important to be aware that self-management may differ from one parent/child dyad to another [28]. Person-centred care highlights the importance of knowing the person behind the patient to engage the person as an active partner in his/her own care and treatment [29]. Guided self-determination (GSD) is an empowerment-based, person-centred, reflection and problem solving method intended to guide the patient to becoming self-determined and developing life skills to manage difficulties in diabetes self-management using worksheets. GSD helps the patient and the health care staff to overcome barriers to empowerment. It has been effective both in individual and group training of adults with type 1 diabetes [30]. Husted et al. have adapted GSD for adolescents and their parents as Guided self-determination-Young (GSD-Y) and there is an ongoing study of adolescents with type 1 diabetes in Denmark [31]. This study will evaluate the effect of an intervention using GSD-Y in groups of adolescents and their parents. It will be carried out at routine outpatient clinics.

### Study aim and hypothesis

The aim of this study is to evaluate whether an intervention with GSD-Y in groups of adolescents starting on insulin pumps and their parents leads to fewer diabetes-related family conflicts, increased perceived health and QoL and improved metabolic control.

We hypothesise that using GSD-Y in groups of adolescents and their parents will increase parental support, and that clarified distribution of responsibility may decrease the negative burden of diabetes, and improve perceived health, QoL and metabolic control among adolescents starting on continuous subcutaneous insulin infusion.

## Methods/design

### Study design and setting

This study is designed as a randomized, controlled, prospective, multicentre study. The study will be conducted at Astrid Lindgren’s Children’s Hospital, Karolinska University Hospital, Stockholm, Sweden and Sachs’ Children and Youth Hospital, Söderjukhuset, Stockholm, Sweden. These hospitals care for all of the children with type 1 diabetes in the region (1200 patients).

### Participants and recruitment

Eighty patients between 12 and 17.99 years who are planning to start CSII will be included. Patients will be excluded if their diabetes has been diagnosed within the past year, HbA1c is < 63 mmol/mol, insulin requirement is < 0.5 IU/kg, if the patient is using continuous glucose monitoring when they start CSII, or if the teenager or their parents have difficulties understanding Swedish. Approximately 4 times per year, information meetings about pump treatment and different insulin pump models will be held at the study sites. Oral and written information about the present study will be given to patients who fulfil the inclusion criteria. Written informed consent for participation in the study will be obtained from the parents and adolescents.

### Randomization

Adolescents and their parents who are willing to participate in the study will be divided consecutively into groups depending on which pump model they have chosen. The groups will then be randomized to either intervention or control. The main reason for group randomization is to make the study feasible within a reasonable time. It is not possible to provide enough staff to start different pump models at the same time. Our experience indicates that there are no differences in distribution between the sexes among those starting CSII; therefore, we have not stratified for gender.

### Intervention

**Figure 1:**
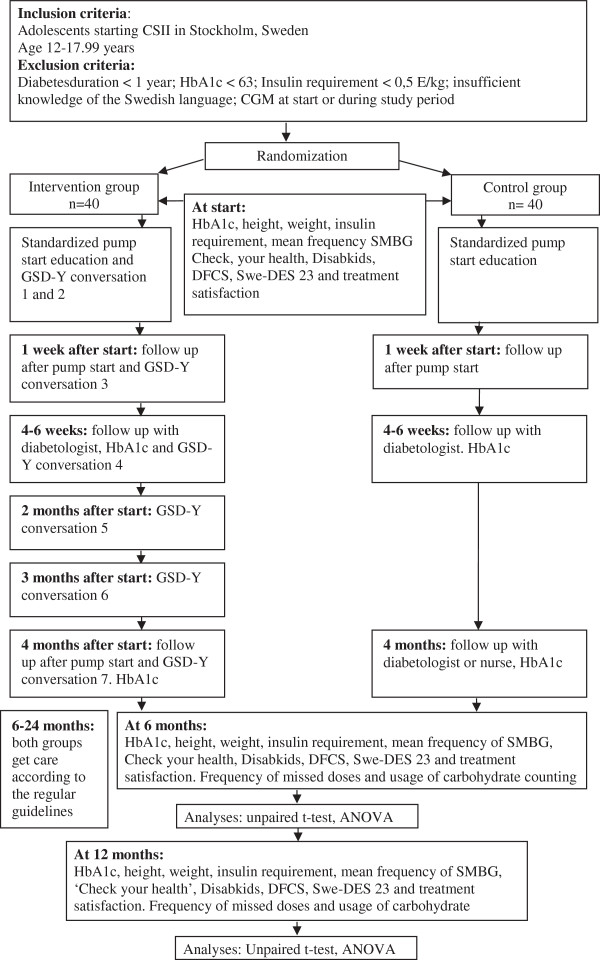
Flow chart of the study.

All adolescents and their parents (both the control group and the intervention group) will receive insulin pump standard start training, including technical skills and how to use carbohydrate counting with CSII. The parents will simulate diabetes by wearing a pump containing saline and test their blood glucose before the child starts insulin pump treatment. The education intervention will be conducted in connection with starting CSII (on three occasions) and on four occasions in the first 4 months after starting CSII. Each session will last 1.5-2 hours. The intervention will be performed by two diabetes nurses (Figure 1). The GSD-Y method will be used with structured reflection sheets. The first reflection sheet will be sent to participants before their first appointment. For subsequent appointments, the reflection sheet for the next meeting will be distributed at that appointment and completed before the next appointment. By filling in the reflection sheet using their own words and drawings, adolescents and their parents systematically explore and express their own experiences and difficulties with diabetes in daily life (Table 1). In the dialogue, different communication models are used, including mirroring, active listening and value-clarifying responses [30,31]. Three diabetes nurses and two dieticians have been trained to use GSD. Together with the creators of GSD and GSD-Y (Zoffmann and Husted) [30,31], we have adjusted GSD-Y so that it fits this study. The worksheets have been translated into Swedish and retranslated by a Danish and Swedish speaking person.

**Table 1:** Overview of reflection sheets

Visit 1 (Start CSII)	**Your life with diabetes from the beginning to now**
	• Written invitation to work together in a new way
	• Two ways of looking at HbA1c
	• Agreement on things to work on
Visit 2 (Start CSII)	**Your life with diabetes from the beginning to now**
	• Important events and periods in your life
	• What do you find difficult at present living with your diabetes?
	• Your plans for changing your way of life
Visit 3 (Start CSII)	**Values and opportunities**
	• Unfinished sentences: needs, values, experiences and opportunities?
Visit 4	**Diabetes in your life**
	• A picture or expression describing your life with diabetes
	• Room for diabetes in your life
	• Shared responsibility between adolescent and parent for diabetes in daily life
	• Common name for a difficulty in your life with diabetes
	• Agreement on things to work on until next visit
Visit 5	**Problem identification and problem solving**
	• Current problem solving
	• Dynamic problem solving
	• Agreement on things to work on until next visit
Visit 6	**Different ways of looking at numbers**
	• Blood glucose (BG) tests and your reasons for checking
	• Actual BG numbers and wishes
	• Your plan for BG regulation in the short and long run.
	• Common name for a difficulty in your life with diabetes
	• Agreement on things to work on until next visit
Visit 7	**Problem identification and problem solving**
	• Current problem solving
	• Dynamic problem solving
	• Solved problems and subjects to continue working on

### Measures and data collection

The data to be collected during this study is shown in Table 2. HbA1c levels will be assessed using a capillary test and analysed using the DCA 2000 apparatus (Siemens Medical Solution Diagnostics, Mölndal, Sweden). Normal reference for children between 6 months and 18 years is 31-38.6 mmol/mol [32]. The mean frequency of bolus doses and percentage doses using carbohydrate counting from the last 2 weeks will be used. The following instruments will be used to evaluate perceived health and QoL: Disabkids, ‘Check your health’, the Diabetes Family Conflict Scale (DFCS) and the Swedish Diabetes Empowerment Scale (Swe-DES 23). Disabkids measures the generic health of children with chronic illnesses and has a specific diabetes module. The Disabkids test has good reliability and validity [33]. ‘Check your health’ measures perceived physical and emotional health, social relationships and general QoL on vertical thermometer scales, ranging from 0 to 100, with 0 indicating low perceived health and QoL. Using the same scales, a person reports what their perceived physical and emotional health, social relationships and QoL would be if they did not have diabetes. The measured difference between, for example, physical health with and without diabetes is defined as the physical burden of diabetes. When the difference results in a positive value, meaning that, for example, imagined physical health without diabetes is reported to be lower than with diabetes, the burden is interpreted as zero. In this study, the marginal values for no burden, low burden, high burden or very high burden will be arbitrary. ‘Check your health’ has been tested on adolescents and adults with diabetes and has shown good reliability and validity [34,35], and it is being validated on parents to young persons with type 1 diabetes. DFCS is the most widely used measure of diabetes-specific family conflicts and has recently been modified [36]. It has been translated and validated on young persons with type 1 diabetes [37]. Swe-DES 23 measures the psychosocial self-efficacy of people with diabetes and has been validated in Swedish for adults. The SWE-DES-23 has four empowerment subscales: goal achievement (alfa: 0.91), self-awareness (alfa: 0.80), stress management (alfa: 0.80), and readiness to change (alfa: 0.68). Cronbach’s alfa-coefficient for the total score was found to be 0.91, demonstrating acceptable reliability and validity [38]. Treatment satisfaction will be measured with one question.

**Table 2:** Data collection

**Before starting CSII**	• HbA1c, height, weight, insulin requirement, mean frequency of self-monitoring of blood glucose (SMBG)
	• ‘Check your health’, Disabkids, DFCS, Swe-DES 23 and treatment satisfaction.
**6 and 12 months after starting CSII**	• Same measures as before starting CSII
	• Frequency of bolus doses and usage of carbohydrate counting

### Statistical analysis and power calculation

To detect a difference of 6 mmol/mol in HbA1c (SD: ± 9.1), at least 37 participants are required in each group (power 80% and alpha 0.05). SD is calculated from the available participants in the actual clinics (n = 160) at study start, mean HbA1c: 74.2 ± 9.1 mmol/mol. Allowing for patient withdrawals and those lost to follow up, 80 patients will be included in the study. All analysis will be conducted on an intention-to-treat basis. The differences between the groups will be analysed using an un-paired t-test with a 95% confidence interval and p < 0.05 considered as significant. A repeated measures ANOVA will be used for before and after comparisons in the groups, using the same confidence interval and level of significance. When testing differences between groups using Disabkids, ‘Check your health’, DFCS and Swe-DES 23, the same statistical analysis will be used.

### Ethical considerations

The Ethical Review Board in Stockholm, Sweden has approved the study (2011/4:6) and the study will be carried out in accordance with the Declaration of Helsinki.

## Discussion

The aim of our study is to provide an educational program in an urban area, and its success depends on how well the study participants represent the population of interest. Previous studies have indicated that children with many diabetes-related conflicts have poorer glycaemic control, and thus, it is important that this group be represented in the study sample [27]. We have chosen to use HbA1c measured at the visit before the planned start of CSII as the inclusion criterion. An alternative, which is common in studies with adults, is to use a mean result from three HbA1c tests taken in the last year. We did not use this latter method as part of the entry criteria because some of the potential patients will have been diagnosed with diabetes for only a short time, and there is a risk of using an HbA1c result from a remission period. GSD is an empowerment-based program, and we have thus chosen to use Swe-DES 23, which is sensitive to change. Previous studies show that unclear responsibility distribution can explain omitted bolus doses and family conflicts, and we will also use DFCS, which is one of the most widely used measures of diabetes-specific family conflict [21,27]. To detect changes in perceived physical and emotional health, social relationships, general QoL and the burden of diabetes, we will use the measures Disabkids and ‘Check your health’ [33-35]. There is a risk that there will be high scores on perceived QoL already at baseline, but it is important to detect both rates of deterioration and improvement. We also want to observe any changes in the burden of diabetes. We will measure treatment satisfaction with one question; we have used this question in previous studies and it has been shown to be sufficient for measuring treatment satisfaction [18]. The intervention will take 4 months and evaluation with measurements of perceived QoL will be performed at baseline, 6 and 12 months to allow comparisons and to determine any changes. Previous research has shown that there is an initial improvement in the first months after the start of treatment using an insulin pump, which later subsides [14,16]. We believe that starting an insulin pump together with an empowerment-based, person-centred education model leads to retention of improved glycaemic control, QoL, responsibility distribution and reduced diabetes-related conflicts in the family.

Self-management in adolescents is inadequate and the distribution of the responsibility in the family regarding diabetes is unclear. Our previous studies have shown, for example, that teens often miss their insulin doses. These results suggest that teenagers do not get proper person-centred care. In addition, there is a lack of evidence in the field, and the intent of this study is to address this gap.

## Abbreviations

CSII: Continuous subcutaneous insulin infusion; MDI: Multiple daily injections; DCCT: The Diabetes Control and Complications Trial; HbA1c: Glycosylated haemoglobin; QoL: Quality of life; GSD: Guided self-determination; GSD-Y: Guided self-determination-Young; SMBG: Self-monitoring of blood glucose; DFCS: The Diabetes Family Conflict Scale; Swe-DES 23: Swedish Diabetes Empowerment Scale.

## Competing interests

The authors declare that they have no competing interests.

## Authors’ contributions

ALB, GV and ALO contributed to the concept and design of the study and they are involved in its implementation. ALB, ALO and JL drafted the manuscript. All authors have read, critically reviewed and approved the final manuscript.

## Pre-publication history

The pre-publication history for this paper can be accessed here:

http://www.biomedcentral.com/1471-2431/13/212/prepub
